# *In vitro* antimicrobial resistance properties of rifapentine and rifampicin are indistinguishable

**DOI:** 10.1128/spectrum.00627-26

**Published:** 2026-05-18

**Authors:** Valeria Barcelli, Bora Shin, Barry Boon Liang Choo, Sarah Leela Dorett, Pablo Bifani

**Affiliations:** 1 Infectious Diseases Translational Research Programme, Department of Microbiology and Immunology, Yong Loo Lin School of Medicine, National University of Singapore 37580, Singapore, Singapore; 2 Lee Kong Chian School of Medicine, Nanyang Technological University, Singapore, Singapore; 3 A*STAR Infectious Diseases Labs, Agency for Science, Technology and Research, Singapore, Singapore; Maryland Department of Health, Baltimore, Maryland, USA

**Keywords:** *Mycobacterium tuberculosis*, rifampicin, rifapentine, resistance, *rpoB*

## Abstract

Tuberculosis (TB) is a major global health threat, with millions of new cases diagnosed annually. Rifampicin is a cornerstone drug in the current standard of care for TB treatment. Recently, rifapentine, an analog of rifampicin, has been considered as an alternative to rifampicin primarily due to its longer half-life and shorter 4-month TB treatment regimens. Despite some encouraging advantages in the clinical setting, the rifamycin resistance mutation frequency and type have yet to be systematically investigated. The mutation identity and frequency could directly impact clinical outcomes and the epidemiology of TB resistance. Our objective was to compare the *in vitro* antimicrobial properties of rifapentine and rifampicin across four *Mycobacterium tuberculosis* strains, namely H37Rv, CDC1551, HN878, and W4, a lineage 2 (Beijing) genotype, and to determine whether resistance differed between the two drugs. We compared the frequency of resistance between rifampicin and rifapentine at various concentrations, and evaluated the frequency of different mutation types in the four chosen TB strains via Sanger sequencing. Overall, no significant differences were observed in the frequency of resistance or mutation identity between rifampicin and rifapentine; however, strain-dependent differences were noted. H37Rv showed a higher frequency of resistance at all concentrations tested in both drugs compared to CDC1551 and the Beijing strains W4 and HN878. The profound overlap in *in vitro* properties between the two antimicrobials provides valuable insight for the ongoing evaluation of rifapentine as a viable alternative to rifampicin in the standard of care for TB treatment.

**IMPORTANCE:**

Recent clinical trials demonstrate that a 4-month regimen combining rifapentine, isoniazid, pyrazinamide, and moxifloxacin is non-inferior to the standard 6-month rifampicin-based regimen. This shorter regimen, with less frequent rifamycin administration, may enhance patient compliance and treatment outcomes. However, two pivotal questions warrant attention before proceeding with a switch to rifapentine-based treatments. First, we must ensure that rifapentine maintains comparable or lower rates of rifamycin resistance, as an increased rate of resistance mutations would undermine the new regimen’s viability. Second, we need to confirm whether current molecular diagnostic tools can accurately detect rifapentine resistance with equivalent sensitivity and specificity. Thus, any rifapentine-specific mutations and their frequency must be identified and incorporated into existing molecular diagnostic platforms if novel. In our investigation, we found that the mutation frequency and repertoire were not significantly different between rifampicin and rifapentine within strains. Current diagnostic methods should be equally sensitive to rifapentine and rifampicin resistance.

## INTRODUCTION

The organism *Mycobacterium tuberculosis* is the etiological agent responsible for tuberculosis (TB), which remains a major global health threat, with millions of new cases diagnosed annually. Implementing antitubercular combination therapy has vastly improved treatment outcomes (1). Rifampicin, a rifamycin, is a fundamental component of antitubercular combination therapy due to its potency, rapid killing kinetics, and bactericidal properties (2, 3). Nevertheless, standard TB regimens last 6 months for active TB and 3 to 4 months for latent TB. This long duration of treatment, along with side effects and other factors, including stigmatization, can result in non-compliance and the possible emergence of drug-resistant TB. Non-adherence prevalence can be as high as 21.2% in some regions (4). Therefore, improving patient compliance is vital in combating the spread of TB and preventing the emergence of multidrug-resistant TB (3).

Rifapentine, an analog of rifampicin, was approved for treating susceptible TB in 1998 and latent TB in 2014 (5, 6). Rifapentine has some pharmacological advantages over rifampicin, including a longer half-life of 13–14 h (7) compared to 3 to 4 h for rifampicin (8), allowing it to be used once weekly to treat latent TB (6, 9, 10). A recent clinical study has shown that a 4-month regimen of daily dosed rifapentine combined with isoniazid, pyrazinamide, and moxifloxacin is non-inferior to our standard 6-month regimen containing rifampicin in treating active tuberculosis (11). The Centers for Disease Control and Prevention (CDC) has recently recommended adopting this 4-month regimen with rifapentine in eligible patients (12). Using shorter treatment regimens has been shown to result in better treatment completion in clinical trials (6, 9, 13). Hence, using rifapentine is an attractive option as it could help improve patient compliance (10).

Rifampicin resistance in *M. tuberculosis* is associated with mutations in the *rpo*B gene encoding the bacterial RNA polymerase beta subunit (14). Over 50 possible mutations associated with rifampicin resistance have been described (15), with mutations 450, 445, and 435 (*Escherichia coli* equivalency can be found in the Supplementary material) accounting for at least 90% of all reported resistance mutations (15, 16). Other rifamycins share the same mechanism of action. Accordingly, mutations in the *rpoB* gene are associated with cross-resistance to rifampicin and other commonly used rifamycins, such as rifapentine, and in some cases, rifabutin (17, 18). However, strains harboring some rifamycin resistance mutations, notably D435V (*E. coli* D516V), remain susceptible to rifabutin (19). With the current recommendation of the CDC calling for a 4-month rifapentine regimen (12, 20), it is likely rifapentine will replace rifampicin as the standard of care for TB treatment in the United States of America. In some circumstances, such as with HIV co-infection, the weekly dosage of rifapentine with isoniazid has been associated with the occurrence of rifamycin mono-resistance (21). Moreover, analogs can be associated with different mechanisms of resistance, such as for amikacin versus kanamycin in TB (22), or the broad-spectrum antibiotics tetracycline and eravacycline (23). Analogs can also have different mechanisms of action in the same or different organisms, as demonstrated by the aminobenzoic acid, sulfonamide, and para-aminosalicylic acid (24). Additionally, the mutation prevention concentration (MPC) has been shown to vary for antimicrobials of the same class, such as fluoroquinolones (25). As such, it is crucial to investigate the microbiological implications of such a shift in treatment regimen. Here, we aim to add to the current literature by comparing the frequency of resistance and the associated mutation types of *M. tuberculosis* when exposed to rifampicin and rifapentine. This insight would aid in analyzing the appropriateness of implementing a 4-month rifapentine regimen as our global standard of treatment.

## RESULTS

### Rifampicin and rifapentine share a similar frequency of resistance in *M. tuberculosis*

**Fig 1:**
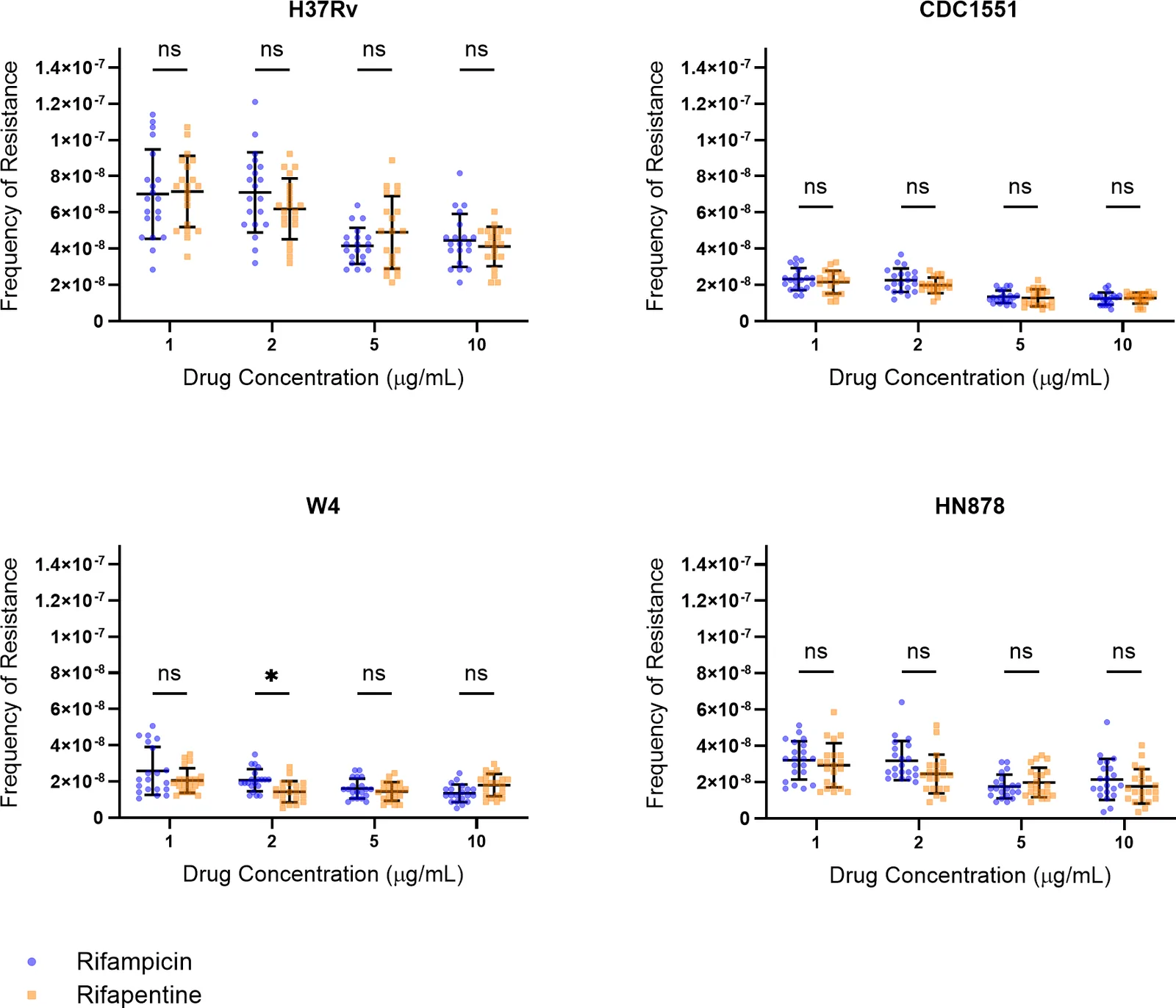
Frequency of resistance of rifampicin and rifapentine at varying concentrations in H37Rv, CDC1551, W4, and HN878. Each data point represents a different technical replicate, and the median ± standard deviation is shown. The data shown in the figure are from a single biological replicate. Asterisks highlight significant differences (*, *P* < 0.05), and “ns” denotes no significance. Statistical significance was determined using two-way ANOVA, followed by Sidak’s multiple comparisons test. Exact values can be found in Table S1.

We measured the frequency at which different strains of *M. tuberculosis* acquire resistance by plating the cultures on 7H11 plates containing 1, 2, 5, and 10 µg/mL of either rifampicin or rifapentine. When comparing the mean frequency of resistance to rifampicin and rifapentine at each concentration tested, our data revealed no significant difference within the strains tested (Fig. 1; Table S1). Statistical significance was determined using two-way ANOVA. However, in W4, a member of lineage 2 (Beijing), a slight difference in the mean frequency of resistance of rifampicin and rifapentine (*P* = 0.0222) was observed at 2 µg/mL. The mean frequency of resistance at 2 µg/mL for rifampicin was 2.07 × 10^−8^, and that of rifapentine was 1.43 × 10^−8^. The mean frequency of resistance for both rifampicin and rifapentine decreases with increased drug concentration in all four strains tested: H37Rv, CDC1551, W4, and HN878.

### The frequency of resistance to both rifampicin and rifapentine is strain dependent

**Fig 2:**
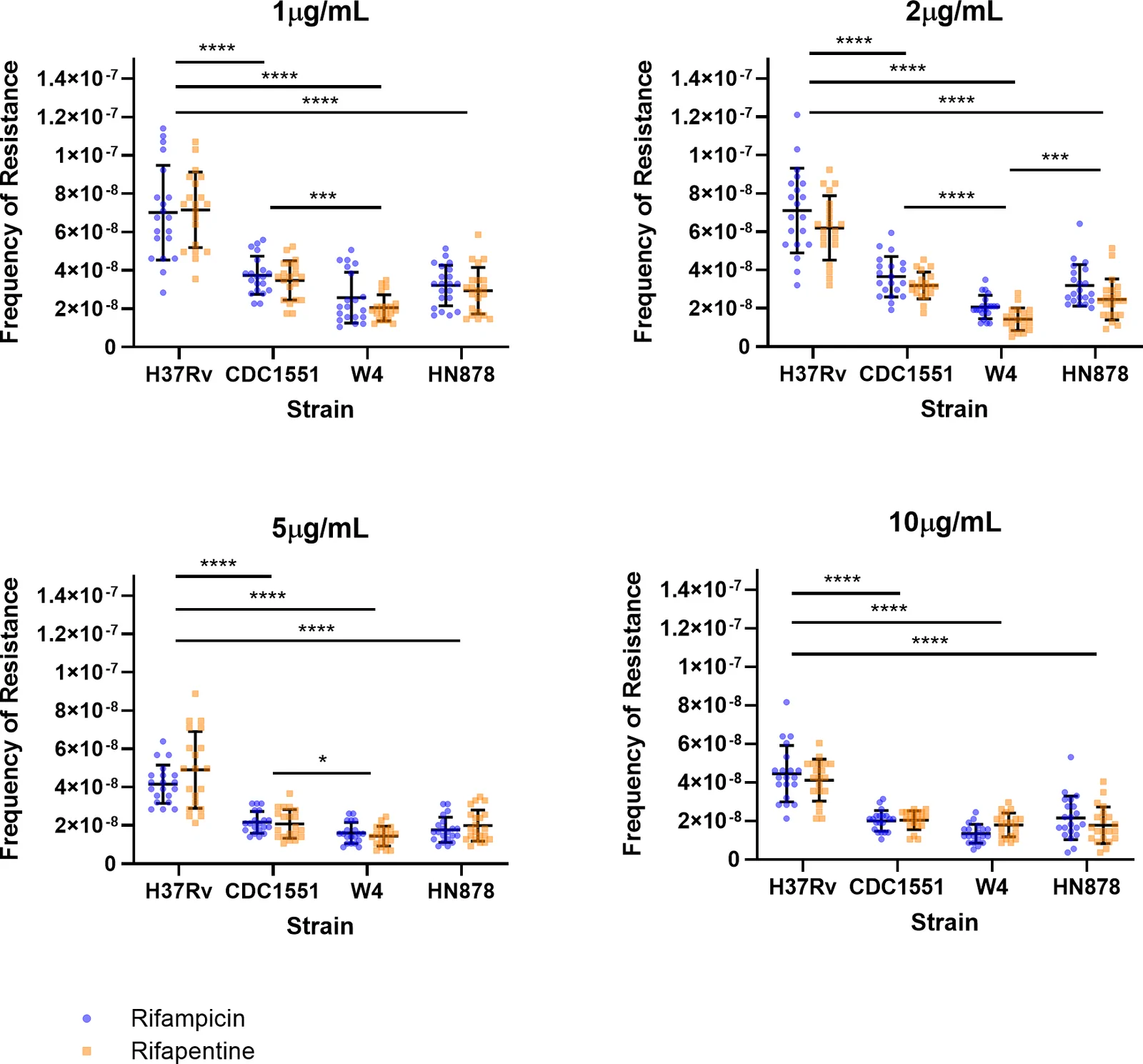
Comparison of the frequency of resistance between H37Rv, CDC1551, W4, and HN878 at different concentrations. Each data point represents a different technical replicate, and the median ± standard deviation is shown. The data shown in the figure are from a single biological replicate. Asterisks highlight significant differences (*, *P* < 0.05; **, *P* < 0.01; ***, *P* < 0.001; ****, *P* < 0.0001). Statistical analysis was done using two-way ANOVA, followed by Tukey’s multiple comparisons test to compare each strain’s mean frequency of resistance.

While the frequency of resistance between rifampicin and rifapentine is similar within the strains, it varies between strains. As shown in Fig. 2, H37Rv has a significantly higher mean frequency of resistance at all four concentrations tested (*P* < 0.0001). In contrast, the lineage 2 (Beijing) strains, W4 and HN878, have the lowest mean frequency of resistance at 1, 2, and 5 µg/mL. The mean frequency of resistance of W4 and HN878 is comparable in all concentrations other than 2 µg/mL (*P <* 0.001). This data is supported by previous findings demonstrating that the frequency of drug resistance is strain-dependent in *M. tuberculosis* (26).

### Rifamycin resistance-conferring mutations are similar when selected on rifampicin and rifapentine

**Fig 3:**
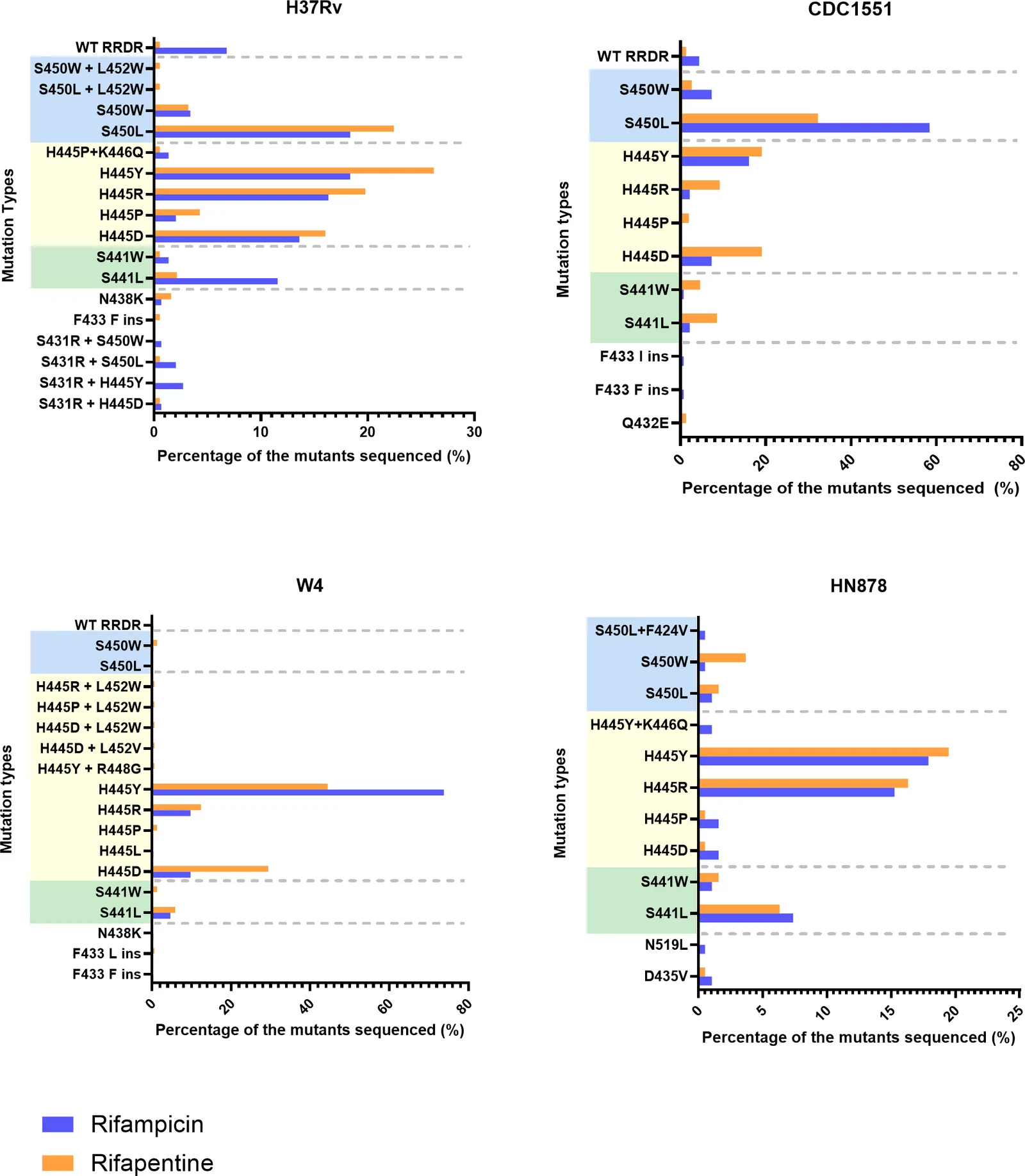
Types of mutations of the RRDR observed and their percentage in H37Rv, CDC1551, W4, and HN878. The exact numbers can be found in Table S2.

**Fig 4:**
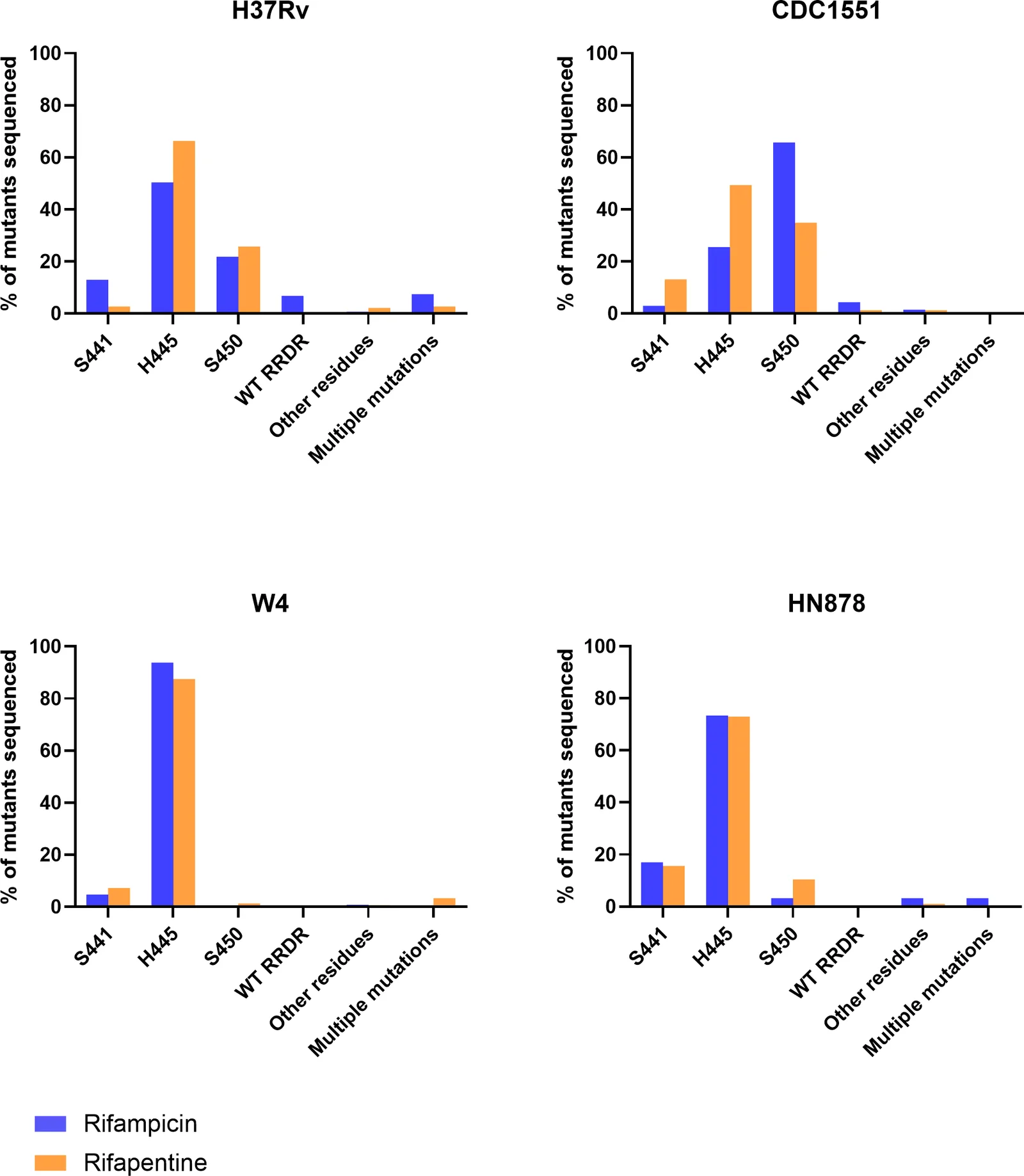
Percentage of single codon substitutions at the most common sites. Exact values can be found in Table S3.

Resistant colonies were systematically picked from plates treated with rifampicin or rifapentine and expanded in the presence of 1 µg/mL of the respective drug. It is worth noting that no false-positive rifamycin-resistant colonies were detected in our study. The rifampicin resistance-determining region (RRDR) was then sequenced to compare the identity of spontaneous mutations selected by the two different drugs. Our screening showed that the mutations observed were the same regardless of the drug used for selection in all four strains tested (Fig. 3; Table S2). However, the distribution of the mutation types observed within each strain differed (Fig. 4). Single amino acid substitutions (SNPs) at codons 445 and 450 were frequently observed in H37Rv and CDC1551, making up 72%–91% of the total mutant population. In contrast, only mutations at codon 445 were frequently observed for the lineage 2 strains, W4 and HN878. In W4, while mutations on codon 445 made up 86%–94% of the total mutant population, only three (one on rifampicin and two on rifapentine) mutations were observed on codon 450, making up 0.3% and 1.3% of the total mutations on rifampicin and rifapentine, respectively. Similarly, in HN878, mutations on codon 445 made up 73% of the mutations on rifampicin and rifapentine. In comparison, mutations on codon 450 accounted for 3% of rifampicin and 10% of rifapentine mutants (Fig. 4; Table S3). Mutations on codon 445 have previously been found to be predominant in the Beijing W lineages reported from the USA, including in the MDR New York W strain associated with nosocomial outbreaks in the early 1990s (27). Together, these data suggest that although the treatment with either rifampicin or rifapentine results in a similar distribution of mutations *in vitro*, the type of mutations observed is strain dependent.

## DISCUSSION

As global efforts are made to shorten the length of tuberculosis treatment regimens, care must be taken to ensure that these novel regimens do not increase the risk of selecting drug-resistant mutants. Our study aims to investigate whether rifapentine’s microbiological effects are comparable to rifampicin *in vitro* and do not lead to a higher rate of resistance (11). Specifically, we compared the frequency of resistance of both drugs in four *M. tuberculosis* strains (H37Rv, CDC1551, and two lineage 2 W-Beijing strains, W4 and HN878) and determined the mutation type for each drug-resistant mutant selected.

Our experiments showed that rifampicin and rifapentine share a similar frequency of resistance *in vitro* in *M. tuberculosis* (Fig. 1). Hence, the use of rifapentine is unlikely to result in an increased risk of the development of rifamycin-resistant variants as compared to the standard rifampicin treatment regimen currently in use. It is important to note that here, the frequency of resistance was determined by calculating the ratio of the average number of mutants observed after selection to the total number of cells plated from a single culture. Other studies looking at the differences in mutation rate instead of frequency use fluctuation tests using methods adapted from Luria and Delbruck (26, 28, 29). While this method is more accurate for comparing strains, it is also more resource-intensive. A systematic determination of the frequency of resistance, as done in our study, is sufficient to compare the microbiological effects of rifampicin and rifapentine.

In our findings, the frequency of resistance appears to be strain dependent. While previous studies have also shown differing frequencies of mutation between different *M. tuberculosis* lineages (26, 30), these studies show that the W-Beijing genotype (lineage 2) has a higher rate of mutation than lineage 4 strains, such as H37Rv and CDC1551, or lineage 3 strains. However, in this study, W4 exhibited a lower resistance frequency than the lineage 4 strain H37Rv. While W4 and HN878 are clinical strains belonging to the W-Beijing genotype, they may not be representative of the entire lineage 2. Different results have been reported in the literature that indicate that there is no difference in the mutation rate in the W-Beijing lineage (lineage 2) compared to lineage 4 (28). These differing results are unsurprising as the W-Beijing strains have evolved independently (31). Furthermore, there have been some notable differences between strains within the W-Beijing family. While the W-Beijing family has been associated with multidrug resistance (27, 32), the W4 strain has explicitly been noted to be generally pan-susceptible (27, 31, 33).

The types of mutations observed in our experiments coincide with those seen in clinical cases, with mutations at codons 450 and 445 being the most common (15, 34), supporting the notion that substituting rifapentine for rifampicin will not lead to the selection of new rifamycin resistance-conferring mutations. From our screening of the mutants selected on rifampicin and rifapentine, we can observe that the types of mutations are the same regardless of the drug used within each strain. Therefore, we can conclude that it is unlikely that rifapentine would select novel mutants *in vitro* or that the MPC would differ greatly between the two.

### Conclusion

In conclusion, our study systematically demonstrated that the frequency of resistance to rifampicin and rifapentine was not significantly different among four *M. tuberculosis* strains: H37Rv, W4, CDC1551, and HN878. Moreover, the resistance-conferring mutations were similar between the two drugs across all tested strains.

## MATERIALS AND METHODS

### Bacterial strains and culture conditions

The *Mycobacterium tuberculosis* strains used were—H37Rv, Beijing strain W4 (33), HN878, and CDC1551 from our laboratory collection. Initial seed stocks of the different *Mycobacterium tuberculosis* strains from our laboratory were prepared by expanding single colonies of each strain, picked from agar plates, into 10 mL of liquid medium. The agar used in this study was Middlebrook 7H11 agar supplemented with oleic acid-albumin-dextrose (Difco Laboratories), and the liquid medium used was Middlebrook 7H9 media supplemented with 10% albumin dextrose saline, 0.05% Tween 80 (Sigma-Aldrich), and 0.2% glycerol (Himedia Laboratories). These are referred to as simply 7H11 and 7H9c in the text. The expanded single colony cultures of each strain were incubated at 37°C until O.D._600_ = 0.6–0.8. Stocks were stored at −80°C in 25% glycerol and thawed at the start of each assay. A biosafety level 3 (BSL-3) facility was used for culturing *M. tuberculosis*.

### Antibiotics

Rifampicin and rifapentine were obtained from Sigma-Aldrich. Stock solutions of 50 mg/mL were made in 100% dimethylsulfoxide (DMSO) before the addition to the culture medium. Final DMSO concentrations were adjusted to 1% in all cases for this study. To prevent exposure to light, drug aliquots were frozen at −20°C in amber 1.5 mL tubes, and drug plates were prepared the day before plating and wrapped in aluminum foil until use the next day.

### Frequency of rifamycin resistance

*M. tuberculosis* strains H37Rv, W4, HN878, and CDC1551 were thawed from 1 mL frozen stocks made previously and grown to an OD_600_ of 0.90 in 100 mL of 7H9c, corresponding to approximately 10^8^ CFU/mL. Each strain was concentrated by centrifugation and resuspension in 7H9c to obtain a final OD_600_ of 10.00. These concentrated cultures of each strain were then plated on 7H11 agar plates containing either rifampicin or rifapentine. The plates contained rifampicin or rifapentine at final concentrations of 1, 2, 5, or 10 µg/mL, corresponding to 1×, 2×, 5×, and 10× the separately determined MIC99 of each compound for our laboratory strains (1 µg/mL for both drugs). Twenty plates were plated for each concentration of both drugs. Additionally, each strain was serially diluted and plated on 7H11 agar without antibiotics for the CFU count. The agar plates were incubated for 6 weeks at 37°C until visible countable colonies appeared. Two biological replicates were carried out.

### Frequency of resistance calculations

The frequency of resistance was calculated using the following formula:


$$\frac{Number\;of\;colonies\;observed}{Total\;number\;of\;cells\;plated}=Frequency\;of\;resistance.$$

The total number of cells plated was obtained from the CFU plating. The frequency of rifamycin resistance was calculated for each strain at each drug concentration.

### Screening mutants observed

Four colonies per plate were systematically picked and each individually grown in 1 mL of 7H9c with 1 µg/mL of rifampicin or rifapentine in 24-well plates. Once grown, the cells were lysed by boiling at 95°C for 30 min. PCR amplification and Sanger sequencing were then done for the *rpoB* gene to screen the mutants. The primers were *rpoB* F1 (5′-ACC GAC GAC ATC GAC CAC-3′) and *rpoB* R1 (5′-CGA ATT GGC CTG TGC CAC-3′).

## SUPPLEMENTAL MATERIAL

Supplemental material spectrum.00627-26-s0001.docx Tables S1 to S3.

ASM does not own the copyrights to Supplemental Material that may be linked to, or accessed through, an article. The authors have granted ASM a non-exclusive, world-wide license to publish the Supplemental Material files. Please contact the corresponding author directly for reuse.
