# Supplementary material for: In vitro antimicrobial resistance properties of rifapentine and rifampicin are indistinguishable
Source: Microbiol Spectr. 2026 May 18;14(7):e00627-26. doi: 10.1128/spectrum.00627-26 (PMC13339808; doi:10.1128/spectrum.00627-26)
Supplement: Supplemental material — Tables S1 to S3. [file spectrum.00627-26-s0001.docx]

**Supplementary Materials**

**In vitro antimicrobial resistance properties of rifapentine and rifampicin are indistinguishable.**

**Valeria Barcelli, Bora Shin, Barry Boon Liang Choo, Sarah Leela Dorett, Pablo Bifani**

**Supplementary Table 1.**

Frequency of resistance to rifampicin and rifapentine in W4, HN878, H37Rv, and CDC1551.

|  |  | **Frequency of Resistance** | | | |
| --- | --- | --- | --- | --- | --- |
|  | **W4** | | **HN878** | **H37Rv** | **CDC1551** |
| **Rifampicin** |  | |  |  |  |
| 1 µg/ml | 2.58E-08 | | 3.14E-08 | 7.01E-08 | 2.32E-08 |
| 2 µg/ml | 2.07E-08 | | 3.13E-08 | 7.10E-08 | 2.26E-08 |
| 5 µg/ml | 1.61E-08 | | 1.82E-08 | 4.15E-08 | 1.34E-08 |
| 10 µg/ml | 1.34E-08 | | 2.19E-08 | 4.46E-08 | 1.24E-08 |
| **Rifapentine** |  | |  |  |  |
| 1 µg/ml | 2.05E-08 | | 3.00E-08 | 7.15E-08 | 2.15E-08 |
| 2 µg/ml | 1.43E-08 | | 2.45E-08 | 6.20E-08 | 1.98E-08 |
| 5 µg/ml | 1.44E-08 | | 1.96E-08 | 4.90E-08 | 1.29E-08 |
| 10 µg/ml | 1.80E-08 | | 1.83E-08 | 4.12E-08 | 1.26E-08 |

**Supplementary Table 2.**

Type of spontaneous mutants observed when selected on rifampicin and rifapentine and their quantity in W4, CDC1551, H37Rv, and HN878.

| **W4** | | | |
| --- | --- | --- | --- |
| *E. coli* numbering of codon mutations | *M. tuberculosis* numbering of codon mutations | Selected on RIF | Selected on RIP |
| F514 F ins | F433 F ins | 1 | 0 |
| F514 L ins | F433 L ins | 0 | 1 |
| N519K | N438K | 1 | 0 |
| S522L | S441L | 12 | 9 |
| S522W | S441W | 0 | 2 |
| H526D | H445D | 25 | 45 |
| H526L | H445L | 1 | 0 |
| H526P | H445P | 0 | 2 |
| H526R | H445R | 25 | 19 |
| H526Y | H445Y | 189 | 68 |
| H526Y + R529G | H445Y + R448G | 0 | 1 |
| H526D + L533V | H445D + L452V | 0 | 1 |
| H526D + L533W | H445D + L452W | 0 | 1 |
| H526P + L533W | H445P + L452W | 0 | 1 |
| H526R + L533W | H445R + L452W | 0 | 1 |
| S531W | S450W | 1 | 2 |
| WT RRDR | WT RRDR | 1 | 0 |
|  | Total | 256 | 153 |

| **CDC1551** | | | |
| --- | --- | --- | --- |
| *E. coli* numbering of codon mutations | *M. tuberculosis* numbering of codon mutations | Selected on RIF | Selected on RIP |
| Q513E | Q432E | 0 | 2 |
| F514 F ins | F433 F ins | 1 | 0 |
| F514 I ins | F433 I ins | 1 | 0 |
| S522L | S441L | 3 | 13 |
| S522W | S441W | 1 | 7 |
| H526D | H445D | 10 | 29 |
| H526P | H445P | 0 | 3 |
| H526R | H445R | 3 | 14 |
| H526Y | H445Y | 22 | 29 |
| S531L | S450L | 80 | 49 |
| S531W | S450W | 10 | 4 |
| WT RRDR | WT RRDR | 6 | 2 |
|  | Total | 137 | 152 |

| **H37Rv** | | | |
| --- | --- | --- | --- |
| *E. coli* numbering of codon mutations | *M. tuberculosis* numbering of codon mutations | Selected on RIF | Selected on RIP |
| S512R + H526D | S431R + H445D | 1 | 1 |
| S512R + H526Y | S431R + H445Y | 4 | 0 |
| S512R + S531L | S431R + S450L | 3 | 1 |
| S512R + S531W | S431R + S450W | 1 | 0 |
| F514 F ins | F433 F ins | 0 | 1 |
| N519K | N438K | 1 | 3 |
| S522L | S441L | 17 | 4 |
| S522W | S441W | 2 | 1 |
| H526D | H445D | 20 | 30 |
| H526P | H445P | 3 | 8 |
| H526R | H445R | 24 | 37 |
| H526Y | H445Y | 27 | 49 |
| H526P+K527Q | H445P+K446Q | 2 | 1 |
| S531L | S450L | 27 | 42 |
| S531W | S450W | 5 | 6 |
| S531L + L533W | S450L + L452W | 0 | 1 |
| S531W + L533W | S450W + L452W | 0 | 1 |
| WT RRDR | WT RRDR | 10 | 1 |
|  | Total | 147 | 187 |

| **HN878** | | | |
| --- | --- | --- | --- |
| *E. coli* numbering of codon mutations | *M. tuberculosis* numbering of codon mutations | Selected on RIF | Selected on RIP |
| D516V | D435V | 2 | 1 |
| N519L | N519L | 1 | 0 |
| S522L | S441L | 14 | 12 |
| S522W | S441W | 2 | 3 |
| H526D | H445D | 3 | 1 |
| H526P | H445P | 3 | 1 |
| H526R | H445R | 29 | 31 |
| H526Y | H445Y | 34 | 37 |
| H526P+K527Q | H445Y+K446Q | 2 | 0 |
| S531L | S450L | 2 | 2 |
| S531W | S450W | 1 | 8 |
| S531L+F505V | S450L+F424V | 1 | 0 |
| WT RRDR | WT RRDR | 0 | 0 |
|  | Total | 94 | 96 |

**Supplementary Table 3**

Summary of the spontaneous mutants observed when selected on rifampicin and rifapentine and their percentage in W4, HN878, H37Rv, and CDC1551

| **W4** | | | | | |
| --- | --- | --- | --- | --- | --- |
| *E. coli* numbering of codon mutations | *M. tuberculosis* numbering of codon mutations | Selected on RIF | Selected on RIP | Selected on RIF % | Selected on RIP % |
| S522 | S441 | 12 | 11 | 4.7% | 7.2% |
| H526 | H445 | 240 | 134 | 93.8% | 87.6% |
| S531 | S450 | 1 | 2 | 0.4% | 1.3% |
| WT RRDR | | 1 | 0 | 0.4% | 0.0% |
| Mutations on other residues | | 2 | 1 | 0.8% | 0.7% |
| Multiple mutations | | 0 | 5 | 0.0% | 3.3% |
|  | Total | 256 | 153 |  |  |

| **HN878** | | | | | |
| --- | --- | --- | --- | --- | --- |
| *E. coli* numbering of codon mutations | *M. tuberculosis* numbering of codon mutations | Selected on RIF | Selected on RIP | Selected on RIF % | Selected on RIP % |
| S522 | S441 | 16 | 15 | 17.0% | 15.6% |
| H526 | H445 | 69 | 70 | 73.4% | 72.9% |
| S531 | S450 | 3 | 10 | 3.2% | 10.4% |
| WT RRDR | | 0 | 0 | 0.0% | 0.0% |
| Mutations on other residues | | 3 | 1 | 3.2% | 1.0% |
| Multiple mutations | | 3 | 0 | 3.2% | 0.0% |
|  | Total | 94 | 96 |  |  |

| **H37Rv** | | | | | |
| --- | --- | --- | --- | --- | --- |
| *E. coli* numbering of codon mutations | *M. tuberculosis* numbering of codon mutations | Selected on RIF | Selected on RIP | Selected on RIF % | Selected on RIP % |
| S522 | S441 | 19 | 5 | 12.9% | 2.7% |
| H526 | H445 | 74 | 124 | 50.3% | 66.3% |
| S531 | S450 | 32 | 48 | 21.8% | 25.7% |
| WT RRDR | | 10 | 1 | 6.8% | 0.5% |
| Mutations on other residues | | 1 | 4 | 0.7% | 2.1% |
| Multiple mutations | | 11 | 5 | 7.5% | 2.7% |
|  | Total | 147 | 187 |  |  |

| **CDC1551** | | | | | |
| --- | --- | --- | --- | --- | --- |
| *E. coli* numbering of codon mutations | *M. tuberculosis* numbering of codon mutations | Selected on RIF | Selected on RIP | Selected on RIF % | Selected on RIP % |
| S522 | S441 | 4 | 20 | 2.9% | 13.2% |
| H526 | H445 | 35 | 75 | 25.5% | 49.3% |
| S531 | S450 | 90 | 53 | 65.7% | 34.9% |
| WT RRDR | | 6 | 2 | 4.4% | 1.3% |
| Mutations on other residues | | 2 | 2 | 1.5% | 1.3% |
| Multiple mutations | | 0 | 0 | 0.0% | 0.0% |
|  | Total | 137 | 152 |  |  |
